# Effect of Counseling Service on Breastfeeding Practice among Saudi Mothers

**DOI:** 10.3390/healthcare11060878

**Published:** 2023-03-17

**Authors:** Alaa AlQurashi, Tariq Wani, Nouf Alateeq, Humariya Heena

**Affiliations:** Research Center, King Fahad Medical City, Riyadh 12231, Saudi Arabia

**Keywords:** attitude, breastfeeding, knowledge, lactation counseling, practice, Saudi Arabia

## Abstract

**Objective:** To assess the knowledge and practice of breastfeeding and the effects of lactation counseling, as a health promotion intervention, on breastfeeding duration and exclusive breastfeeding (EBF) among Saudi women. **Methods:** In this quasi-experimental design study, 664 mothers attending a women’s hospital from January 2017 to December 2018 were interviewed. Women were allocated into two groups, control and intervention groups, based on received lactation counseling. Interviews were performed using a structured questionnaire collecting data on the sociodemographic characteristics, knowledge, attitude and practice of breastfeeding mothers. A chi-squared test was used to determine the level of significance on breastfeeding practices among two groups. Propensity score matching was presented to control confounders, as women cannot be randomly assigned to lactation counseling. **Results:** Of 664 women, 592 were Saudi nationals, and the majority of mothers were literate (96.2%). A significantly higher number of mothers in the consultation group were employed as compared with the non-consultation group (*p* = 0.015). One third (33.3%) of the women practiced EBF, while 39.8% preferred mixed feeding for the first six months of the infant’s life. The consultation group demonstrated a significantly higher response rate in their knowledge on the benefits of breastfeeding in babies (increased intelligence; *p* < 0.05) and mothers (breast engorgement; *p* = 0.004), colostrum and its importance (*p* = 0.027) and effective breastfeeding practices (initiate breastfeeding within 30 min after birth (*p* = 0.01), baby needs 10–20 min between each feed (*p* = 0.009), breastfeeding should last for 6 months (*p* = 0.01)) compared with the non-consultation group. The age of weaning (5.3 ± 2.8 vs. 5.9 ± 3.2 months) was similar across both the groups. However, “the intended duration of BF” was higher in the non-consultation group, and the difference was statistically significant (*p* = 0.002). The mean weight and length of the baby at follow-up were similar in both groups, with no statistical significance. The duration of exclusive breastfeeding among two groups was not statistically significantly different (8.7 ± 6.9 vs. 8.1 ± 7.1 weeks). Mothers in the two groups were satisfied with their breastfeeding experience. The mean scores lie within the range of 4.2 to 5.0. Baby age (month), mother LoE, mother job and type of BF were controlled for, and the propensity-score-matched 62.5% sample from both the groups yielded the same results. **Conclusions:** Breastfeeding women in our study showed a fair knowledge of EBF. However, the duration of actual EBF was very short, and the counseling intervention showed no impact on EBF in our study population. We recommend interventions that are tailored to the needs of this population, while identifying factors that improve breastfeeding practice among mothers.

## 1. Introduction

Breastfeeding is the ideal, unique and natural method for nourishing the developing infant during the first few months of life. Breast milk contains the complete range of nutrients required by infants for healthy growth and development [1]. Breastfeeding has both short- and long-term impacts on the health, nutrition and development of the child and mother’s health [2]. Appropriate breastfeeding practices prevent child morbidity and mortality from diarrhea, respiratory illness, otitis media, gastroenteritis, necrotizing enterocolitis, sudden infant death syndrome, obesity and hypertension [3]. Additionally, it reduces the mother’s risk of developing breast cancer, ovarian cancer and type 2 diabetes mellitus and improves birth spacing [3]. Breastfeeding also reduces healthcare and feeding costs. Not breastfeeding or suboptimal breastfeeding is associated with lower intelligence and economic losses of about USD 302 billion annually or 0.49% of the world gross national income [2]. The World Health Organization (WHO) and UNICEF advocate exclusive breastfeeding (EBF) for the first six months after birth, and recommend breastfeeding be continued for two years or longer together with nutritionally adequate complementary foods [1]. Breastfeeding exclusively for a longer duration is positively associated with the healthy development of the child’s neuro-system, protection from neuro-diseases and better cognitive abilities [4]. One study shed light on the contribution of counseling services provided by a healthcare provider in improving the rate of EBF even if provided through phone call follow-ups [5]. A systematic review showed that counseling service has proven to be effective in increasing any breastfeeding initiation. The odds ratio for any initiation was 1.35 higher than no initiation with a confidence interval of 95%. It also proves its benefit in increasing median and mean duration. In addition, interventions involving lactation consultants and counselors raise the odds ratio for any breastfeeding versus not breastfeeding by 1.76 from 1.20 to 2.57 with a 95% CI. Lastly, the exclusive breastfeeding appeared to have a positive impact in infants up to one month old but showed no statistical effect in infants between 3 and 6 months. Another meta-analysis concluded that e IBCLC has the potential to improve breastfeeding [6].

In recent quasi-experimental research, prenatal lactation counseling delivered to primigravidae promoted breastfeeding practices and breast engorgement [7].

Despite demonstrated benefits, the practice of EBF in the first six months of age has declined, including in the Kingdom of Saudi Arabia (KSA) [8]. Women are willing to breastfeed and a high percentage of them initiate breastfeeding, but they prefer mixed feeding and tend to introduce formula milk early, which results in a cessation of breastfeeding [9,10]. Several factors were found to affect the decision to breastfeed [11]. In the KSA, studies have cited the perception of inadequate breast milk that could result from a lack of breastfeeding knowledge as the most frequent reason for early breastfeeding discontinuation [12,13], as well as distinct cultural and social norms, which might indicate a different approach to adaption for intervention compared to elsewhere. Professional support has been documented to be effective in increasing women’s confidence, resulting in a higher level of EBF [14]. However, in the KSA, no data have been reported on the outcomes for such intervention [15]. 

The Ministry of Health (MoH) KSA encourages baby-friendly hospitals as per the WHO standards for achieving the Millennium Development Goals (MDGs) of EBF in hospitals [16]. Hence, counselor service for mothers is provided throughout the antenatal and postnatal period at several hospitals under the MoH [17]. This service is usually carried out immediately after birth (i.e., in the first three days and up to one week). The effectiveness is achieved when this service is continued through a follow-up process, either by follow-up telephone calls or by arranging a follow-up visit with the mother [18]. It is, therefore, crucial to examine the impact of such lactation services implemented to tackle the problem of the low prevalence of breastfeeding in the KSA and propose relevant courses of action to change the status quo. The outcomes are important, as maternal and child health is a priority area with the provision of support for the most effective strategy to promote EBF [19]. 

The present study was, therefore, conducted to assess the effects of a lactation counselor as a health promotion intervention on breastfeeding duration and EBF in Saudi women. 

## 2. Methods 

### 2.1. Study Design

This quasi-experiment was conducted among women at a tertiary care hospital in Riyadh, KSA, from January 2017 to December 2018. The study was conducted to determine if lactation counseling made a difference in the level of mothers’ knowledge on breastfeeding practice, duration, completeness, EBF and satisfaction among treatment and control groups. Women who gave birth at the hospital and attended at least 8 appointments were eligible to receive the lactation service and assigned to the intervention group. Women who gave birth outside but attended less than 8 visits at the study hospital prior to giving birth are not eligible for the lactation service, and were assigned to the control group.

The Institutional Review Board approved the study protocol with reference number IRB00010471. Written informed consent was obtained from all subjects before inclusion in the study.

#### Inclusion and Exclusion Criteria

We included women ≥ 18 years of age with normal pregnancies, meaning that they did not face pregnancy medical complications such as deep vein thrombosis in pregnancy, high blood pressure (hypertension) in pregnancy, itching and intrahepatic cholestasis of pregnancy or severe vomiting in pregnancy [20]. Babies included in the study were born within the normal period at 36 to 44 weeks of gestation and received normal newborn care according to WHO essential newborn care, which includes “*immediate care at birth (delayed cord clamping, thorough drying, assessment of breathing, skin-to-skin contact, early initiation of breastfeeding),thermal care, resuscitation when needed, support for breastmilk feeding, nurturing care, infection prevention, assessment of health problems, recognition and response to danger signs and timely and safe referral”* [21]. In contrast, women who were excluded were women with complicated pregnancy and delivery mentioned previously, such as an unusual placenta position, fetal growth less than the 10th percentile for gestational age (fetal growth restriction) and rhesus (Rh) sensitization—a potentially serious condition that can occur when your blood group is Rh negative and your baby’s blood group is Rh positive.

### 2.2. Subject Selection and Data Collection

With regard to assigning the intervention and control groups, we could not assign subjects randomly to each group due to eligibility restrictions (counseling provided only to women who gave birth at the study hospital).

A trained research assistant checked the medical records of antenatal women undergoing regular checkups during pregnancy at the hospital. 

The original list had 1500 women. Then, women were divided into two groups; consultation and non-consultation groups, based on the status of receiving the counseling services. By using a simple random sampling technique, a total of 664 women were approached. 

Breastfeeding practice was assessed using a study questionnaire within 24 h from receiving lactation counseling. Both the intervention group and control group were approached either during hospital visits or via phone calls.

#### 2.2.1. Breastfeeding Knowledge Questionnaire

Data were collected by trained nurses working with the lactation team. They used a structured questionnaire that was developed based on the literature of previously published research [22,23]. Content validity was evaluated by breastfeeding educators, lactation specialists and research experts (two each). Revisions were made as per the recommendations and certain irrelevant questions were deleted. A pilot study was conducted on the pre-final version of the questionnaire to determine whether the questions were understandable, and the questionnaire was revised based on the comments from the participants. The pilot study found Cronbach’s alpha to be 0.90.

The questionnaire assessing knowledge of breastfeeding comprised 38 items, including general knowledge, colostrum, advantages to mothers and babies, effective feeding method, duration of feeding, complementary feeding and problems with breastfeeding. The scores of the test ranged from 0 to 38, with higher scores indicating better knowledge. Each item had categorical responses of “yes”, “no” or “I do not know”. A correct answer was scored as “1,” whereas incorrect or missing answers were assigned “0”. 

Breastfeeding practices were assessed with the questions regarding duration of breastfeeding, breastfeeding completeness and duration of EBF. Moreover, breastfeeding satisfaction was phrased as “how do you feel about the experience of having breastfed your baby?”, assessed using a five-point scale where 1 meant “very unfavorable” and 5 meant “very favorable”. 

#### 2.2.2. Description of the Lactation Services 

Certified lactation consultants from the International Board Certified Lactation Consultant (IBCLC) are responsible for helping women with their breastfeeding and childcare questions and concerns. The counseling process usually begins when women come for the 8th antenatal visits; the doctor refers her to one lactation counselor. The lactation services provided in Saudi Arabia are only conducted at health care facilities; no home visits are offered by lactation consultants. Postpartum counseling services are provided through phone call follow-ups, or during the 8-week appointment. 

The lactation services included two types of consultation—educational and practical sessions. For the education part, a trained specialist educated mothers during a face-to-face meeting providing her with all the required knowledge and evidence-based information regarding the mother’s milk production and the positive effect of breastfeeding on her newborn. Specialists also provided mothers with educational materials including a brochure and a booklet. These contained information regarding the importance of breastfeeding, duration of feeding and feeding instructions.

In addition, they were invited to a practical session where mothers tried breastfeeding on a model and baby doll under supervision, to learn the best technique for breastfeeding a newborn. Mothers were followed up with through phone calls in different periods after delivery (1st week, 3rd week, 16th week) with a face-to-face routine appointment during pregnancy. A total of at least three consultations were conducted pre- and post-delivery. 

#### 2.2.3. Sample Size Calculation

The prevalence of EBF in the KSA is 20% [15]. Bearing in mind the expected population of 18,000, establishing a margin of error equivalent to 5% at a 95% confidence interval and 80% power, the estimated sample size on whom counseling had to be implemented was at least 332 mothers. An equal number of mothers who did not receive EBF counseling were chosen as a comparison group.

### 2.3. Statistical Analysis

Descriptive and analytic statistics were used to analyze the study findings. Descriptive statistics such as mean, median, mode and standard deviation were used for numerical variables, and percentages and proportions were used for categorical variables. 

The chi-squared test was used to determine the association between the variables with a level of significance set at *p* < 0.05. Other relevant associations determining breastfeeding practice were also analyzed using chi-squared tests. The missing data were not taken into consideration throughout the analysis. The statistical analysis was performed using Statistical Program for Social Sciences (SPSS) software package version 20.

The baseline socio-demographic characteristics in both the consultation and non-consultation groups were matching, and differences were not significant. Moreover, the propensity-score-matched 62.5% sample from both the groups yielded the same results. 

Binary logistic regression of respiratory failure controlling for participant age (yr), baby age (month), mother LoE, mother job and type of BF predicted the respective probabilities, for which the mean (SD) was 0.5048 (0.0895) for the 664 cases loaded in the modal. Calipher width was derived by multiplying 0.6 by the SD of the probabilities, and the derivative score 0.05 was thereupon used for the propensity score matching (PSM) tolerance analysis. The exact matching fetched 205 cases from the consultation group and 209 cases from the non-consultation group.

## 3. Results

### 3.1. Sociodemographic and Breastfeeding Characteristics of the Participants

Of the 664 subjects enrolled for breastfeeding counseling services, 331 (49.8%) were in the consultation group and 333 (50.2%) in the non-consultation group. All mothers had normal pregnancies and were followed up with post-delivery at the hospital at 3 months and 6 months postpartum. 

In total, 592 (89.2%) of the study participants were Saudi nationals, which was nearly equal between the consultation and non-consultation groups (294 (88.8%) vs. 298 (89.5%), *p* = 0.782). The mean age of the participants in each group was 31.6 ± 5.8 years and 31 ± 5.9 years. 

The difference in the level of education (LoE) among participants in the consultation group compared with the non-consultation group was not significant (*p* = 0.302). Similarly, there was no significant difference between the two groups regarding the husband’s highest LoE (*p* = 0.303). A significantly higher proportion of mothers were employed in the consultation group compared with the non-consultation group (*p* = 0.015). Furthermore, 97 (29.1%) mothers in the consultation and 123 (37.5%) in the non-consultation groups practiced EBF, but the difference was not significant (*p* = 0.073).

The age of weaning (5.3 ± 2.8 vs. 5.9 ± 3.2 months) was similar across both the groups (Table 1). However, “the intended duration of BF” difference between the two groups was statistically significant (*p* = 0.002). The mean weight and length of the baby at follow-up were similar in both the groups, with no statistical significance. The duration of exclusive breastfeeding among the two groups was not statistically significantly different (8.7 ± 6.9 vs. 8.1 ± 7.1 weeks) (Table 2). 

The baseline socio-demographic characteristics in both the consultation and non-consultation groups were matching, and the difference was not significant. Moreover, the propensity-score-matched 62.5% sample from both the groups yielded the same results (Table 1 and Table 2).

### 3.2. Breastfeeding Knowledge

Among the knowledge questions regarding lactation benefits to babies, the correct response rate in the consultation group exceeded that in the non-consultation group for all knowledge statements, e.g., for ‘breastfeeding has a significant effect on intelligence in children’, 198 (62.7%) responded correctly in the consultation group compared to 158 (49.8%) in the non-consultation group (*p* < 0.05) (Table 3). 

Similarly, for knowledge of lactation benefits in mothers, the correct response rate in the consultation group was more than in the non-consultation group. However, significant differences were observed across only two of the five statements, ‘Breastfeeding reduces the chances of breast engorgement’ (202 (68.5%) vs. 187 (60.3%)) and ‘Mothers who breastfeed are less likely to develop breast cancer’ (185 (63.8%) vs. 162 (53.5%)) (Table 3). 

The knowledge regarding lactation benefits of colostrum in the consultation group was higher than in the non-consultation group with a significant difference (*p* = 0.027). 

The knowledge of mothers regarding the benefits of effective breastfeeding was higher in the consultation group compared with the non-consultation group for statements ‘Children sleep better when they are fed enough’ (169 (61.0%) vs. 178 (59.5%) *p* = 0.907). For other factors such as ‘Children gain weight when they are fed effectively’ and ‘Correct positioning’, the response rate was low in the consultation group compared with the non-consultation group, 207 (73.4%) vs. 244 (81.3%), *p* = 0.006 and 173 (63.6%) vs. 190 (65.7%), *p* = 0.173, respectively. The proportionate difference, however, for both the factors was not significant.

For the knowledge regarding duration of feeding, the response rate in the consultation group was higher than in the non-consultation group for the following statements: “Breastfeeding should begin within 30 min after birth,” (168 (63.2%) vs. 162 (56.8%), *p* = 0.010), and “The baby needs 10 to 20 min between each feed,” (140 (62.8%) vs. 100 (48.3%), *p* = 0.009).

In addition, the knowledge regarding complementary feeding was significantly higher in the consultation group than in the non-consultation group for “The added food should begin in the sixth month,” (174 (74.0%) vs. 160 (62.0%), *p* = 0.010). The response rate for “Mixed feed,” was comparable in both the groups.

The response rate for statements between the two groups was not found to be statistically significant. Binary logistic regression of respiratory failure controlling for participant age (yr), baby age (month), mother LoE, mother job and type of BF predicted the respective probabilities, for which the mean (SD) was 0.5048 (0.0895) for the 664 cases (Table 3). 

The experience of the mothers with breastfeeding was evaluated using a five-point satisfaction scale, which was represented by the mean ± SD score.

“The breastfeeding experience” mean score for consultation group was 4.3 ± 1.2 and that for non-consultation group was 4.4 ± 1.1. The mean scores lie within the range of 4.2 to 5.0, which shows that mothers were “extremely satisfied” with their breastfeeding experience and the difference was not statistically significant between the two groups.

The mean response score for “How likely you are to re-experience breastfeeding if you have another child?” among the consultation group was 3.7 ± 1.5 and that for the non-consultation group was 3.9 ± 1.5. The mean scores lie within the range of 3.4 to 4.2, which shows that the mothers would “most likely” breastfeed another child.

Using PSM, each consultation unit was matched with a non-consultation unit of similar knowledge characteristics, The propensity score is presented in Table 3. 

## 4. Discussion

This quasi-experimental study assessed the level of knowledge on breastfeeding practice, duration, completeness, EBF and satisfaction among pregnant Saudi women at a tertiary care hospital in Riyadh, KSA. In this study, the majority of the participants were literate, employed and had a good knowledge of the benefits of effective breastfeeding. 

Upon testing participants’ BF knowledge using a questionnaire, the reported correct answers from both groups were as follows: the consolation group reported a higher proportion of correct answers for several questions such as the correct time to introduce solid food and the right estimation of the BF duration. In contrast, the non-consultation group reported a higher proportion of correct answers to more questions, for instance the correct positioning, the effect of BF on infant sleeping and the correct time of when to start BF. However, we could not conclude that the BF counseling increased participants’ knowledge; it was not possible to make a statistical comparison because there is no baseline for comparison.

The reported answers highlight the limitation of the BF consultation service in Saudi Arabia during the study time as there is no home visit provided and counseling is either not provided or through phone calls, which is more feasible but has limitations such as the inability to personally assess the home environment, which could be a barrier to BF initiation, incapability of visually demonstrating information that is explained in person and, finally, the difficulty in following up with unresponsive mothers, which could be less effective in comparison to home visits. Home visits have the advantages that it is possible to initiate failed assessment and visually demonstrate necessary information. However, some of the phone call barriers in the study could be overcome by, for example, using the list from medical records to confirm status of receiving lactation counseling. The data collection is carried out by experts not from the lactation team to avoid reporting bias due to emotional relations between mothers and the counselor. In conclusion, the current BF counseling service is incompatible with the recent international findings that showed the positive impact of BF home visits, ideally three to seven visits [24].

Mothers thought that breastfeeding could have a significant impact in terms of improving children’s intelligence, and could also prevent the risk of developing breast cancer among breastfeeding women. Although the majority of mothers rated their breastfeeding experience to be extremely satisfactory, the intended duration of breastfeeding was significantly shorter among the non-consultation group compared with the consultation group.

In our study, EBF was practiced by a third of the women, while an equal number preferred mixed feeding for the first six months of the infant’s life. This suggests that, despite their high level of education, the prevalence of EBF was extremely low and suboptimal, while mixed feeding was the predominant mode of feeding in our study population. This was in line with previous studies where the percentage of EBF ranged from as low as 1.2% to 31.4% [11,25,26]. 

The most common reason cited for discontinuing breastfeeding was breast milk insufficiency. The issue of perceived inadequate breast milk can be managed through proper breastfeeding awareness programs and certified lactation professional support. In some countries, this service is offered through maternal and child health services, while in others it is as a supplementary approach towards health promotion organizations. Professional support is usually provided to mothers after giving birth and the personnel responsible for such service are usually a lactation specialist [27,28].

Maternal occupation did affect the duration and frequency of breastfeeding per day though it was not a barrier to prevent mothers from breastfeeding [29]. Taken together, this points to the fact that there is an increased need to continuously counsel women on the benefits of breastfeeding. 

Breast milk is the natural source of nutrition for newborn infants, providing essential nutrients for healthy growth and development [30]. Evidence suggests that healthcare support including doctors, lactation nurses, women, infants and children (WIC) peer counselors and skin-to-skin care encouragement by nurses are important factors that govern early initiation and sustained breastfeeding practices [31]. The results of the present study concur with previous studies where postpartum breastfeeding education/support showed a positive impact on the duration of EBF [32]. Studies have shown that interventions that provide extra support show an increase in the length of time women continued to breastfeed and the length of time women breastfed without introducing any other type of liquid or food [33]. 

In previous studies, interventions were performed with only postnatal support, consisting of either antenatal or postnatal support intervention or with only antenatal support. However, a systemic review found no beneficial effect of interventions containing an antenatal component on the duration of breastfeeding [34]. In our study, we preferred a single postnatal support intervention that was performed only once postpartum and on the first follow-up visit either at 3 or 6 months after delivery. Such an approach using single intervention could be easier and more cost-effective than repeated interventions [34]. The core strength of this intervention is that it considers different needs of individuals, so the type of support could vary and also the depth of this support could vary in new mothers [35]. Moreover, in our study, we included face-to-face support as it is associated with a larger treatment effect than supportive intervention provided over the phone [36]. 

Most of the mothers who had received the consultation service from the hospital were aware of the benefits of colostrum and knew that colostrum is the first liquid of the mother’s milk that is thick and yellow with a viscous consistency, as compared with the mothers in non-consultation group. This is similar to a study conducted in Pakistan, where more than a quarter of mothers did not know about the health benefits of colostrum and the ones who knew had received guidance from healthcare professionals [37]. 

There are barriers that could have limited the effectiveness of the study intervention, such as cultural barriers. Acknowledging the conservative nature of the community, the process of taking permission from mothers to allow health care providers to visit home is not feasible. Furthermore, on a policy level, there is no policy established yet to enforce the implementation of the home service.

Our study has several limitations. The sample size of this research might not be representative of the population of the whole country. The study does not count the first one-week period after delivery, as the follow-up was carried out only after the first week postpartum. This could have some impact on the results due to a lag period of one week, which is crucial for newborn babies and lactating mothers. Additionally, the eligibility criteria could be a potential source of bias, since women who gave birth at the hospital and attended at least eight appointments, and may be different from women who gave birth outside but attended fewer than eight.

## 5. Conclusions

Our study in breastfeeding women showed their fair knowledge of EBF. However, the duration of actual EBF was short and the counseling intervention showed no impact on EBF in our study population. There is a need for focused interventions on improving the self-efficacy of Saudi mothers and to encourage public health professionals to shift the focus of the health promotion program from “need to breastfeed” to “how to breastfeed”. Our study will provide the necessary impetus to the Ministry of Health in planning and designing a health promotion strategy that is aligned with the culture of KSA and strongly advocates the availability of the postpartum services for breastfeeding counseling for mothers who deliver either at institutions or at primary healthcare centers. We strongly recommend that support for breastfeeding mothers should be tailored to the setting and the needs of the population group. Further research is needed to identify the aspects of support that are the most effective, and focus on interventions to improve breastfeeding practice among Saudi mothers.

## Figures and Tables

**Table 1:** Sociodemographic characteristics.

						PSM Sociodemographic Characteristics		
Characteristics	Description	Consultation	No Consultation	Total, *N* (%)	*p*-Value	Consultation	No Consultation	*p* Value
	Sample Size	333 (50.2)	331 (49.8)	664 (100.0)		205 (49.5)	209 (50.5)	
Nationality	Saudi	294 (88.8)	298 (89.5)	592 (89.2)	0.782	178 (86.8)	183 (87.6)	0.824
	Non-Saudi	37 (11.2)	35 (10.5)	72 (10.8)		27 (13.2)	26 (12.4)	
Participant Age (yr)	min–max	19–46	19–47	19–47	0.347	19–46	19–47	0.542
	Mean ± SD	31.2 ± 5.9	31.6 ± 5.8	31.4 ± 5.8		31.4 ± 6	31.8 ± 5.7	
	Median (P25, P75)	31 (27, 35)	31 (27, 36)	31 (27, 36)		31 (27, 36)	31 (28, 36)	
Number of children	min–max	1–8	1–9	1–9	0.445	1–8	1–9	0.827
	Mean ± SD	3 ± 1.6	3.1 ± 1.8	3.0 ± 1.7		3 ± 1.7	3 ± 1.9	
	Median (P25, P75)	3 (2, 4)	3 (2, 4)	3 (2, 4)		3 (2, 4)	3 (1, 4)	
Mother’s LoE	Illiterate	10 (3.2)	14 (4.4)	24 (3.8)	0.302	7 (3.4)	8 (3.8)	0.166
	Intermediate or less	26 (8.4)	34 (10.8)	60 (9.6)		18 (8.8)	24 (11.5)	
	Secondary	115 (37.2)	101 (32.0)	216 (34.6)		78 (38.0)	56 (26.8)	
	Postgraduate	21 (6.8)	32 (10.1)	53 (8.5)		17 (8.3)	24 (11.5)	
	University	137 (44.3)	135 (42.7)	272 (43.5)		85 (41.5)	97 (46.4)	
Husband’s LoE	Illiterate	4 (1.4)	7 (2.3)	11 (1.9)	0.303	2 (1.0)	7 (3.5)	0.053
	Intermediate or less	10 (3.4)	20 (6.7)	30 (5.1)		5 (2.5)	13 (6.5)	
	Secondary	69 (23.3)	69 (23.2)	138 (23.2)		41 (20.8)	51 (25.6)	
	Postgraduate	46 (15.5)	49 (16.4)	95 (16.0)		35 (17.8)	26 (13.1)	
	University	167 (56.4)	153 (51.3)	320 (53.9)		114 (57.9)	102 (51.3)	
Mother’s employment	House worker	158 (60.3)	209 (70.1)	367 (65.5)	0.015	133 (64.9)	152 (72.7)	0.085
	Employed	104 (39.7)	89 (29.9)	193 (34.5)		72 (35.1)	57 (27.3)	
Type of breastfeeding	Exclusive	97 (29.1)	123 (37.5)	220 (33.3)	0.073	70 (34.1)	67 (32.1)	0.896
	Partial	140 (42.0)	123 (37.5)	263 (39.8)		78 (38.0)	81 (38.8)	
	Bottle	96 (28.8)	82 (25.0)	178 (26.9)		75 (27.8)	61 (29.2)	

All values are *N*(%) unless otherwise stated. Abbreviations: BF: breastfeeding; EBF: exclusive breastfeeding; LoE: level of education, *N*: total sample size; n: total number of subjects in a subset.

**Table 2:** Breastfeeding-related characteristics between the two groups.

					PSM Breastfeeding-Related Characteristics		
Characteristics	Description	Consultation	No Consultation	*p*-Value	Consultation	No Consultation	*p* Value
	Sample Size	333 (50.2)	331 (49.8)		205 (49.5)	209 (50.5)	
Age of children at weaning (months)	Mean ± SD	5.3 ± 2.8	5.9 ± 3.2	0.034	6 ± 3.3	5.3 ± 2.8	0.021
	Median (min, max)	6 (3, 8)	5 (3, 7)		6 (3, 8)	5 (3, 7)	
Intended duration of BF (weeks)	Mean ± SD	39.6 ± 26	47.4 ± 26.1	0.002	52.9 ± 25.2	39.2 ± 25	<0.001
	Median (min, max)	52 (24, 52)	52 (20, 52)		52 (52, 52)	52 (24, 52)	
Weight of the baby at follow-up (g)	Mean ± SD	5 ± 1.9	4.9 ± 2.1	0.373	4.7 ± 2.2	5.2 ± 2.1	0.077
	Median (min, max)	4.5 (3, 6.6)	5 (3.3, 6.5)		4 (2.9, 6.8)	5.3 (3.2, 7)	
Length of the baby at follow-up (g)	Mean ± SD	59.3 ± 8.8	58.6 ± 8.3	0.608	57.7 ± 9.9	59.6 ± 11	0.054
	Median (min, max)	59 (55, 65)	59 (55, 64)		59 (54, 61)	60 (55, 65)	
EBF duration (weeks)	Mean ± SD	8.7 ± 6.9	8.1 ± 7.1	0.581	10.0 ± 8.2	8.22 ± 7.7	0.244
	Median (min, max)	8 (2, 12)	8 (4, 12)		8 (4, 13)	4 (4, 13)	

**Table 3:** Comparison of knowledge regarding breastfeeding between the two groups.

						PSM Comparison of Knowledge Regarding Breastfeeding		
Characteristics	Description	Consultation	No Consultation	Total	*p*-Value	Consultation	No Consultation	*p* Value
	Sample Size	333 (50.2)	331 (49.8)	664 (100.0)		205 (49.5)	209 (50.5)	
Type of BF	Exclusive	123 (37.5)	97 (29.1)	220 (33.3)	0.073	70 (34.1)	67 (32.1)	0.896
	Partial	123 (37.5)	140 (42.0)	263 (39.8)		78 (38.0)	81 (38.8)	
	Bottle	82 (25.0)	96 (28.8)	178 (26.9)		57 (27.8)	61 (29.2)	
BF reduces the risk of respiratory infection	No	52 (16.1)	54 (16.9)	106 (16.5)	0.137	40 (19.8)	34 (17.0)	0.47
	Yes	203 (62.8)	178 (55.8)	381 (59.3)		123 (60.9)	118 (59.0)	
	I do not know	68 (21.1)	87 (27.3)	155 (24.1)		39 (19.3)	48 (24.0)	
BF increases the child’s intelligence	No	56 (17.7)	75 (23.7)	131 (20.7)	0.005	36 (18.5)	44 (22.1)	0.01
	Yes	198 (62.7)	158 (49.8)	356 (56.2)		131 (67.2)	106 (53.3)	
	I do not know	62 (19.6)	84 (26.5)	146 (23.1)		28 (14.4)	49 (24.6)	
BF reduces diarrhea	No	60 (19.0)	45 (14.3)	105 (16.7)	0.105	37 (19.0)	25 (12.8)	0.21
	Yes	195 (61.7)	190 (60.5)	385 (61.1)		116 (59.5)	121 (61.7)	
	I do not know	61 (19.3)	79 (25.2)	140 (22.2)		42 (21.5)	50 (25.5)	
BF reduces allergy	No	41 (13.1)	40 (12.9)	81 (13.0)	0.585	26 (13.3)	17 (8.8)	0.306
	Yes	227 (72.3)	216 (69.5)	443 (70.9)		134 (68.7)	144 (74.6)	
	I do not know	46 (14.6)	55 (17.7)	101 (16.2)		35 (17.9)	32 (16.6)	
Teeth and gum	No	32 (10.1)	21 (6.7)	53 (8.4)	0.304	23 (11.7)	12 (6.2)	0.003
	Yes	236 (74.4)	240 (76.7)	476 (75.6)		133 (67.9)	161 (82.6)	
	I do not know	49 (15.5)	52 (16.6)	101 (16.0)		40 (20.4)	22 (11.3)	
BF stimulates uterine contractions	No	29 (10.0)	47 (15.0)	76 (12.6)	0.096	18 (10.1)	26 (13.5)	0.063
	Yes	187 (64.3)	201 (64.2)	388 (64.2)		121 (68.0)	141 (73.4)	
	I do not know	75 (25.8)	65 (20.8)	140 (23.2)		39 (21.9)	25 (13.0)	
Breastfeeding reduces the chances of breast engorgement	No	53 (18.0)	48 (15.5)	101 (16.7)	0.004	40 (22.1)	27 (14.3)	0.119
	Yes	202 (68.5)	187 (60.3)	389 (64.3)		119 (65.7)	132 (69.8)	
	I do not know	40 (13.6)	75 (24.2)	115 (19.0)		22 (12.2)	30 (15.9)	
Mothers who breastfeed are less likely to develop breast cancer	No	46 (15.9)	49 (16.2)	95 (16.0)	0.014	29 (16.5)	27 (15.0)	0.177
	Yes	185 (63.8)	162 (53.5)	347 (58.5)		118 (67.0)	109 (60.6)	
	I do not know	59 (20.3)	92 (30.4)	151 (25.5)		29 (16.5)	44 (24.4)	
Mothers who breastfeed are less likely to have osteoporosis	No	35 (12.2)	28 (9.5)	63 (10.8)	0.087	22 (12.4)	17 (9.7)	0.702
	Yes	204 (71.1)	197 (66.8)	401 (68.9)		119 (67.2)	121 (68.8)	
	I do not know	48 (16.7)	70 (23.7)	118 (20.3)		36 (20.3)	38 (21.6)	
Mothers who breastfeed are likely to lose weight faster	No	37 (13.4)	44 (15.5)	81 (14.5)	0.667	19 (11.2)	30 (18.2)	0.161
	Yes	182 (65.7)	186 (65.7)	368 (65.7)		114 (67.1)	106 (64.2)	
	I do not know	58 (20.9)	53 (18.7)	111 (19.8)		37 (21.8)	29 (17.6)	
Colostrum is an important part of the mother’s milk and is thick, viscous and yellow- colored	No	33 (11.9)	23 (7.7)	56 (9.7)	0.027	21 (12.5)	10 (5.5)	0.067
	Yes	192 (69.3)	196 (65.3)	388 (67.2)		115 (68.5)	136 (74.3)	
	I do not know	52 (18.8)	81 (27.0)	133 (23.1)		32 (19.0)	37 (20.2)	
Mothers who breastfeed are less likely to have jaundice in babies	No	84 (34.9)	82 (36.1)	166 (35.5)	0.512	64 (38.3)	59 (33.5)	0.506
	Yes	87 (36.1)	71 (31.3)	158 (33.8)		56 (33.5)	58 (33.0)	
	I do not know	70 (29.0)	74 (32.6)	144 (30.8)		47 (28.1)	59 (33.5)	
Children gain weight when they are fed effectively	No	40 (14.2)	32 (10.7)	72 (12.4)	0.066	29 (16.9)	21 (11.4)	0.001
	Yes	207 (73.4)	244 (81.3)	451 (77.5)		121 (70.3)	157 (84.9)	
	I do not know	35 (12.4)	24 (8.0)	59 (10.1)		22 (12.8)	7 (3.8)	
Correct positioning during BF helps to achieve effective breastfeeding	No	59 (21.7)	71 (24.6)	130 (23.2)	0.173	44 (26.5)	41 (23.3)	0.003
	Yes	173 (63.6)	190 (65.7)	363 (64.7)		95 (57.2)	125 (71.0)	
	I do not know	40 (14.7)	28 (9.7)	68 (12.1)		27 (16.3)	10 (5.7)	
Children sleep better when they are fed enough	No	74 (26.7)	81 (27.1)	155 (26.9)	0.907	50 (29.1)	45 (24.5)	0.384
	Yes	169 (61.0)	178 (59.5)	347 (60.2)		98 (57.0)	118 (64.1)	
	I do not know	34 (12.3)	40 (13.4)	74 (12.8)		24 (14.0)	21 (11.4)	
BF should begin within 30 min after birth	No	29 (10.9)	58 (20.4)	87 (15.8)	0.01	18 (10.7)	31 (17.8)	0.097
	Yes	168 (63.2)	162 (56.8)	330 (59.9)		106 (63.1)	109 (62.6)	
	I do not know	69 (25.9)	65 (22.8)	134 (24.3)		44 (26.2)	34 (19.5)	
The baby needs 10 to 20 min between each feed	No	54 (24.2)	73 (35.3)	127 (29.5)	0.009	43 (26.9)	57 (35.0)	0.141
	Yes	140 (62.8)	100 (48.3)	240 (55.8)		95 (59.4)	79 (48.5)	
	I do not know	29 (13.0)	34 (16.4)	63 (14.7)		22 (13.8)	27 (16.6)	
Complementary feeding	The added food should begin in the sixth month	174 (74.0)	160 (62.0)	334 (67.7)	0.01	116 (78.9)	108 (71.5)	0.241
Mixed feeding	Mothers may confuse breastfeeding with artificial feeding at the start of supplementary food	127 (54.3)		262 (54.1)	0.346	43 (28.9)	38 (26.2)	0.872
			135 (54.0)			80 (53.7)	80 (55.2)	
						26 (17.4)	27 (18.6)	
BF should be continued up to 2 years	No	50 (23.5)	61 (30.3)	111 (26.8)	0.212	34 (22.5)	49 (30.8)	0.196
	Yes	128 (60.1)	115 (57.2)	243 (58.7)		90 (59.6)	89 (56.0)	
	I do not know	35 (16.4)	25 (12.4)	60 (14.5)		27 (17.9)	21 (13.2)	
EBF should last for 6 months	No	36 (15.3)	66 (25.6)	102 (20.7)	0.01	18 (12.2)	29 (19.2)	0.241
	Yes	174 (74.0)	160 (62.0)	334 (67.7)		116 (78.9)	108 (71.5)	
	I do not know	25 (10.6)	32 (12.4)	57 (11.6)		13 (8.8)	14 (9.3)	
Do you know the difference between predominant BF and exclusive BF?	No	68 (29.1)	62 (24.8)	130 (26.9)	0.346	43 (28.9)	38 (26.2)	0.872
	Yes	127 (54.3)	135 (54.0)	262 (54.1)		80 (53.7)	80 (55.2)	
	I do not know	39 (16.7)	53 (21.2)	92 (19.0)		26 (17.4)	27 (18.6)	
BF is affected by breast size	No	54 (21.6)	61 (23.1)	115 (22.4)	0.904	36 (23.4)	38 (24.2)	0.966
	Yes	152 (60.8)	159 (60.2)	311 (60.5)		95 (61.7)	97 (61.8)	
	I do not know	44 (17.6)	44 (16.7)	88 (17.1)		23 (14.9)	22 (14.0)	
If the nipples are not prominent (inverted nipples), the mother cannot breastfeed her child	No	55 (24.4)	50 (20.7)	105 (22.5)	0.546	42 (30.0)	27 (19.1)	0.069
	Yes	119 (52.9)	139 (57.4)	258 (55.2)		76 (54.3)	82 (58.2)	
	I do not know	51 (22.7)	53 (21.9)	104 (22.3)		22 (15.7)	32 (22.7)	
Breastfeeding reduces the chances of breast engorgement	No	26 (10.9)	17 (6.8)	43 (8.8)	0.188	20 (13.1)	17 (11.5)	0.858
	Yes	165 (69.0)	171 (68.7)	336 (68.9)		105 (68.6)	101 (68.2)	
	I do not know	48 (20.1)	61 (24.5)	109 (22.3)		28 (18.3)	30 (20.3)	
EBF should last for 6 months	No	41 (16.5)	57 (22.5)	98 (19.5)	0.212	27 (17.4)	23 (15.5)	0.858
	Yes	172 (69.1)	159 (62.8)	331 (65.9)		113 (72.9)	112 (75.7)	
	I do not know	36 (14.5)	37 (14.6)	73 (14.5)		15 (9.7)	13 (8.8)	
Massage reduces inflammation and swelling of the breast	No	45 (19.1)	55 (22.9)	100 (21.1)	0.494	29 (20.3)	32 (22.9)	0.869
	Yes	147 (62.6)	138 (57.5)	285 (60.0)		88 (61.5)	83 (59.3)	
	I do not know	43 (18.3)	47 (19.6)	90 (18.9)		26 (18.2)	25 (17.9)	
Child should be given water after each feed	No	77 (33.3)	76 (32.1)	153 (32.7)	0.247	52 (36.4)	41 (30.4)	0.179
	Yes	118 (51.1)	110 (46.4)	228 (48.7)		71 (49.7)	64 (47.4)	
	I do not know	36 (15.6)	51 (21.5)	87 (18.6)		20 (14.0)	30 (22.2)	
Belching indicates fullness	No	65 (25.9)	83 (32.7)	148 (29.3)	0.246	38 (24.4)	48 (32.0)	0.32
	Yes	140 (55.8)	129 (50.8)	269 (53.3)		92 (59.0)	81 (54.0)	
	I do not know	46 (18.3)	42 (16.5)	88 (17.4)		26 (16.7)	21 (14.0)	

Abbreviations: BF: breastfeeding; EBF: exceptional breastfeeding.

## Data Availability

Data available on request due to restrictions related to women’s’ privacy and cultural consideration.
